# Supplementary material for: n-3 Polyunsaturated Fatty Acids Modulate LPS-Induced ARDS and the Lung–Brain Axis of Communication in Wild-Type versus Fat-1 Mice Genetically Modified for Leukotriene B4 Receptor 1 or Chemerin Receptor 23 Knockout
Source: Int J Mol Sci. 2023 Aug 31;24(17):13524. doi: 10.3390/ijms241713524 (PMC10487657; doi:10.3390/ijms241713524)
Supplement: Supplementary file 1 [file ijms-24-13524-s001.zip › ijms-2557055-supplementary.pdf]

Supplementary Figures and Tables

n-3 Polyunsaturated Fatty Acids Modulate LPS-Induced ARDS and the Lung–Brain Axis of Communication in Wild-Type versus Fat-1 Mice Genetically Modified for Leukotriene B4 Receptor 1 or Chemerin Receptor 23 Knockout

Jessica Hernandez [1,†], Julia Schäffer [1,2,†], Christiane Herden [3], Fabian Johannes Pflieger [1], Sylvia Reiche [2], Svenja Körber [3], Hiromu Kitagawa [4], Joelle Welter [1], Susanne Michels [5], Carsten Culmsee [5,6], Jens Bier [2], Natascha Sommer [2], Jing X. Kang [7], Konstantin Mayer [8], Matthias Hecker [2,*] and Christoph Rummel [1,6,*]

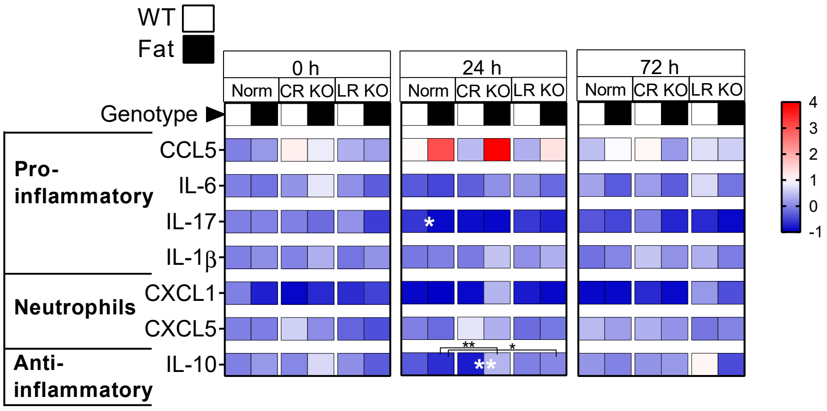

Figure S1. Changes in inflammatory mediators in the liver following intratracheal LPS-induced ARDS. Multiplex cytokine measurements from homogenized liver tissue assessing pro-inflammatory cytokines: chemokine (C-C motif) ligand 5 (CCL5), interleukin (IL)-6, IL-17 and IL-1β; neutrophil chemoattractants: chemokine (C-X-C motif) ligand (CXCL)1 and CXCL5; and the anti-inflammatory cytokine: IL-10. Mice deficient in chemerin receptor 23 (CR KO) or leukotriene B4 receptor (LR KO) as well as unmodified mice (Norm) bred on a wild-type (WT) or transgenic omega-3 (n-3) synthesizing *fat-1* (Fat) background received an intratracheal (i.t.) instillation with lipopolysaccharide (LPS, 10μg) and were sacrificed at 0 h, 24 h or 72 h p.i. An impact of n-3 PUFAs and the RvE1 receptors CR and LR were most prominent at 24 h p.i. Please note that only few significant differences between groups were observed. The average fold change for each sample set were normalized to WT-Norm 0 h and presented as a value on a heat map. n = 3-5 per group after outlier exclusions. *$p<0.05$, **$p<0.01$.

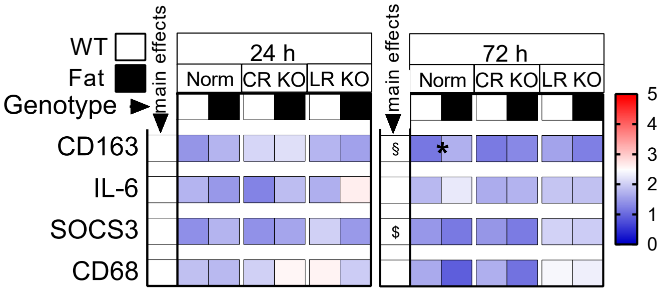

Figure S2: Changes in hypothalamic inflammatory mediator mRNA expression following intratracheal LPS-induced ARDS. The hypothalamus was analyzed for expression of markers for macrophage: CD163; cytokine: interleukin (IL)6; signaling pathway: suppressor of cytokine signaling (SOCS)3; and microglia activation: CD68. Mice deficient in chemerin receptor 23 (CR KO) or leukotriene B4 receptor (LR KO) as well as unmodified mice (Norm) were bred on a wild-type (WT) or transgenic omega-3 (n-3) synthesizing *fat-1* (Fat) background received an intratracheal (i.t.) instillation with lipopolysaccharide (LPS, 10µg) and were sacrificed at 24 h or 72 h p.i. Preliminary analysis at 24 h and 72 h p.i. showed minor impacts of n-3 PUFAs and the RvE1 receptors CR and LR at 72 h p.i. but only for CD163 and SOCS3. The average relative expression for each sample set is presented as a value on a heat map. n = 5 per group for all groups except for 72 h WT-Norm (n = 4). Main effect of receptor: $ Norm vs. LR KO. Main effects of n-3 PUFAs: § Norm vs. CR KO. *p<0.05.

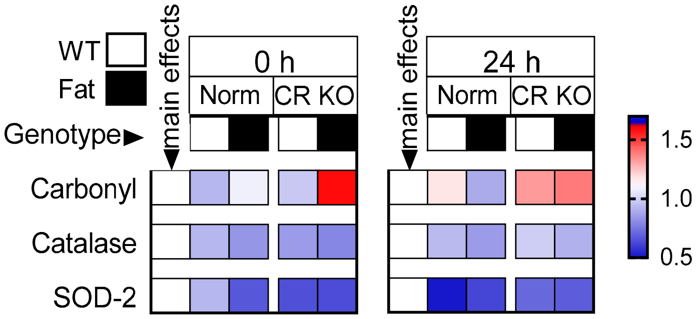

Figure S3. Oxidative stress marker detection in the hypothalamus following intratracheal LPS-induced ARDS by a protein carbonylation assay and Western blot. The hypothalamus was analyzed for markers for oxidative stress, namely, protein carbonylation (Carbonyl) as well as protein levels of catalase (Cat) and superoxide dismutase (SOD)2. Mice deficient in chemerin receptor 23 (CR KO) as well as unmodified mice (Norm) were bred on a wild-type (WT) or transgenic n-3 synthesizing *fat-1* (Fat) background received an intratracheal (i.t.) instillation with lipopolysaccharide (LPS, 10µg) and were sacrificed at 0 h or 24 h p.i. An impact of omega-3 (n-3) PUFAs and the RvE1 CR were not observed. The average fold change for each sample set is normalized to WT-Norm 0 h and is presented as a value on a heat map. n = 5 per group for all groups except for Cat 0 h WT-CR KO (n = 4), SOD2 0 h Fat-CR KO (n = 4), Cat and SOD2 24 h WT-CR KO (n = 4). No significant changes.

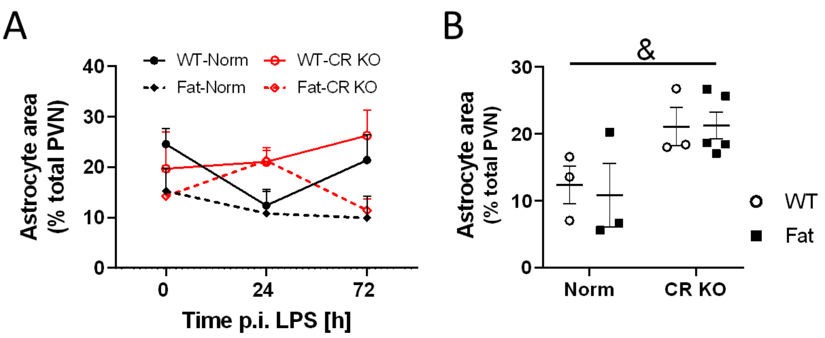

Figure S4. Astrocyte immunoreactivity in the hypothalamus following intratracheal LPS-induced ARDS. The hypothalamus was analyzed at the level of the paraventricular nucleus (PVN) for the percentage of astrocyte area per image unit using the astrocytic marker GFAP. Mice deficient in chemerin receptor 23 (CR KO) as well as unmodified mice (Norm) were bred on a wild-type (WT) or transgenic n-3 synthesizing *fat-1* (Fat) background received an intratracheal (i.t.) instillation with lipopolysaccharide (LPS, 10µg) and were sacrificed at 0 h, 24 h or 72 h p.i. (A). A main effect of the RvE1 CR was observed at 24 h p.i. where deficiency in CR increased the astrocyte area in the PVN (B). n = 3 per group for all groups except for 0 h WT-Norm (n = 4), 0 h Fat-Norm (n = 4), 0 h WT-CR KO (n = 2), 24 h Fat-CR KO (n = 5), 72 h WT-Norm (n = 2), 72 h Fat-Norm (n = 4). Statistics were only performed at the 24 h time point when n = >3 per group. & main effect of the CR. *p<0.05.

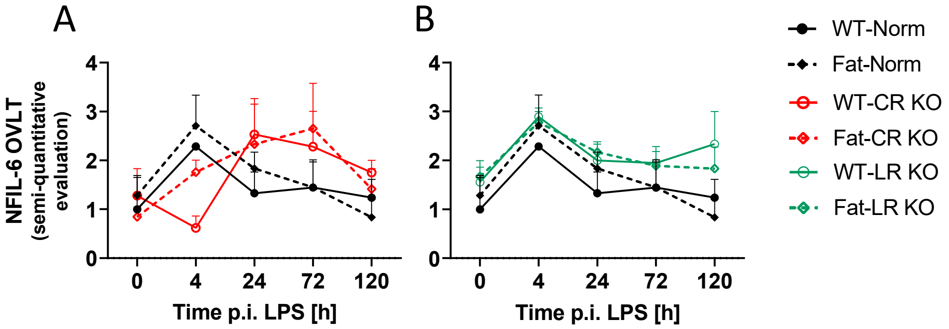

Figure S5. NF-IL6 immunoreactivity in Norm, CR KO and LR KO mice at the level of the OVLT over time after intratracheal LPS-induced ARDS. Sections of the brain were analyzed at the level of the vascular organ of lamina terminalis (OVLT) for nuclear factor interleukin 6 (NF-IL6) immunoreactivity on a scale ranging from 0-4 (A, B). Unmodified mice (Norm) were compared to mice deficient in chemerin receptor 23 (CR KO; A) or leukotriene B4 receptor (LR KO; B) bred on a wild-type (WT) or transgenic omega-3 (n-3) synthesizing *fat-1* (Fat) background received an intratracheal (i.t.) instillation with lipopolysaccharide (LPS, 10µg) and were sacrificed at 0 h, 4 h, 24 h, 72 h and 120 h p.i. Sections were analyzed by immunofluorescence staining using an antibody for NF-IL6. Immunoreactivity was assessed using semi-quantitative evaluation as previously applied [65]. n = 1-5 per group. Statistics were not performed due to low ‘n’ numbers.

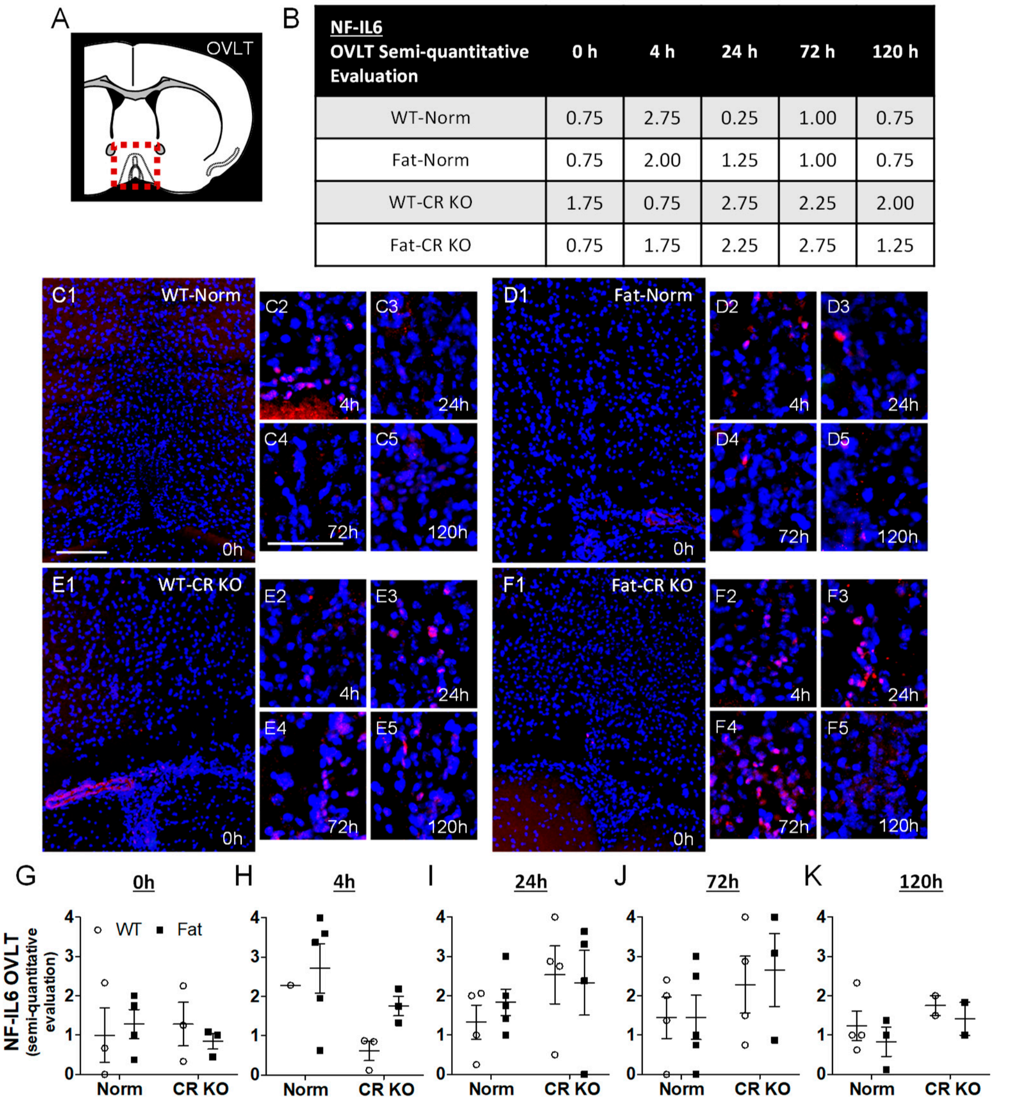

NF-IL6 OVLT Semi-quantitative Evaluation	0 h	4 h	24 h	72 h	120 h
WT-Norm	0.75	2.75	0.25	1.00	0.75
Fat-Norm	0.75	2.00	1.25	1.00	0.75
WT-CR KO	1.75	0.75	2.75	2.25	2.00
Fat-CR KO	0.75	1.75	2.25	2.75	1.25

Figure S6. Intratracheal LPS-induced ARDS NF-IL6 immunoreactivity at the level of the OVLT in Norm compared to CR KO mice is not significantly altered at a specific time point. Sections of the brain were analyzed at the level of the vascular organ of the lamina terminalis (OVLT; A) by semi-quantitative evaluation for nuclear factor interleukin 6 (NF-IL6; red, C-F) immunoreactivity on a scale ranging from 0-4 (B). Unmodified mice (Norm; C) were compared to mice deficient in the chemerin receptor 23 (CR KO; E, F) bred on a wild-type (WT; C, E) or transgenic omega-3 (n-3) synthesizing *fat-1* (Fat; D, F) background received an intratracheal (i.t.) instillation with lipopolysaccharide (LPS, 10µg) and were sacrificed at 0 h, 4 h, 24 h, 72 h and 120 h p.i. WT- / Fat-Norm mice were compared to WT- / Fat-CR KO groups at each time point (G-K). No differences were observed between groups. Statistics were only performed when n = >3 per group. Von Willebrand factor (green; C-F) depicts brain vasculature. DAPI (blue; C-F) visualizes the surrounding tissue. n = 1-5 per group. Scale bar in C1 = 100 µm and is representative for C1, D1, E1 and F1; Scale bar in C4 = 50 µm and is representative for C2-5, D2-5, E2-5 and F2-5.

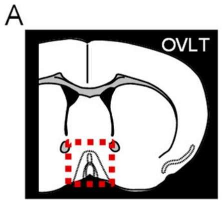

B

**NF-IL6 OVLT Semi-quantitative Evaluation**	**0 h**	**4 h**	**24 h**	**72 h**	**120 h**
WT-Norm	0.75	2.75	0.75	1.00	0.75
Fat-Norm	0.75	2.00	1.25	1.00	0.75
WT-LR KO	1.75	3.00	2.00	2.00	2.25
Fat-LR KO	1.75	2.75	2.25	2.00	1.75

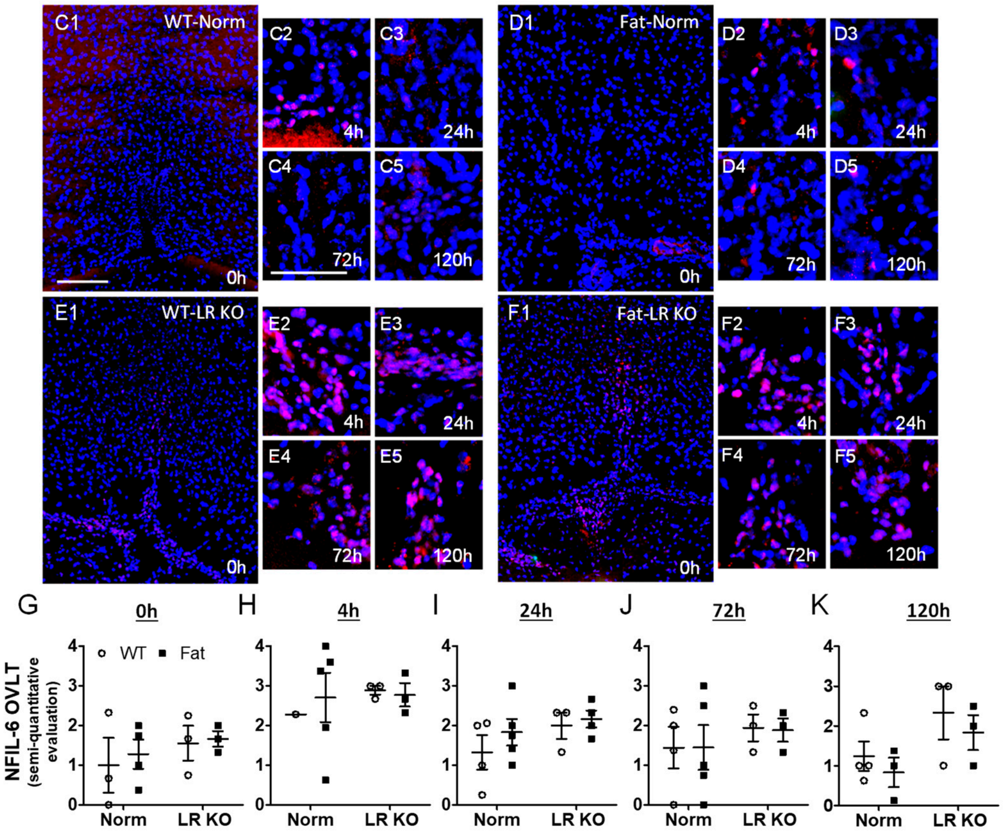

Figure S7: Intratracheal LPS-induced ARDS NF-IL6 immunoreactivity in the OVLT in Norm compared to LR KO mice is not significantly altered at a specific time point. Sections of the brain were analyzed at the level of the organum vasculosum lamina terminalis (OVLT; A) by semi-quantitative evaluation for nuclear factor interleukin 6 (NF-IL6; red, C-F) immunoreactivity on a scale ranging from 0-4 (B). Unmodified mice (Norm; C) were compared to mice deficient in the leukotriene B4 receptor (LR KO; E, F) bred on a wild-type (WT; C, E) or transgenic omega-3 (n-3) synthesizing *fat-1* (Fat; D, F) background received an intratracheal (i.t.) instillation with lipopolysaccharide (LPS, 10µg) and were sacrificed at 0 h, 4 h, 24 h, 72 h and 120 h p.i. WT- / Fat-Norm groups were compared to WT- / Fat-LR KO groups at each time point (G-K). No differences were observed between groups. Statistics were only performed when n = >3 per group. Von Willebrand factor (green; C, D) depicts brain vasculature. Myeloperoxidase (green; E, F) visualizes neutrophils. DAPI (blue; C-F) visualizes the surrounding tissue. Please note that data on WT-Norm and Fat-Norm controls is displayed again (Figure S6) for comparison. n = 1-5 per group. Scale bar in C1 = 100 µm and is representative for C1, D1, E1 and F1; Scale bar in C4 = 50 µm and is representative for C2-5, D2-5, E2-5 and F2-5.

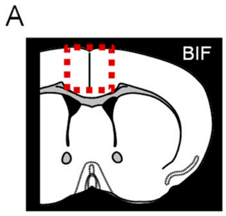

B

NF-IL6 BIF Semi-quantitative Evaluation	0 h	4 h	24 h	72 h	120 h
WT-Norm	0.75	2.75	0.75	1.00	0.75
Fat-Norm	0.75	2.00	1.25	1.00	0.75
WT-LR KO	1.75	2.25	1.75	0.75	1.25
Fat-LR KO	1.75	2.25	1.75	1.00	1.75

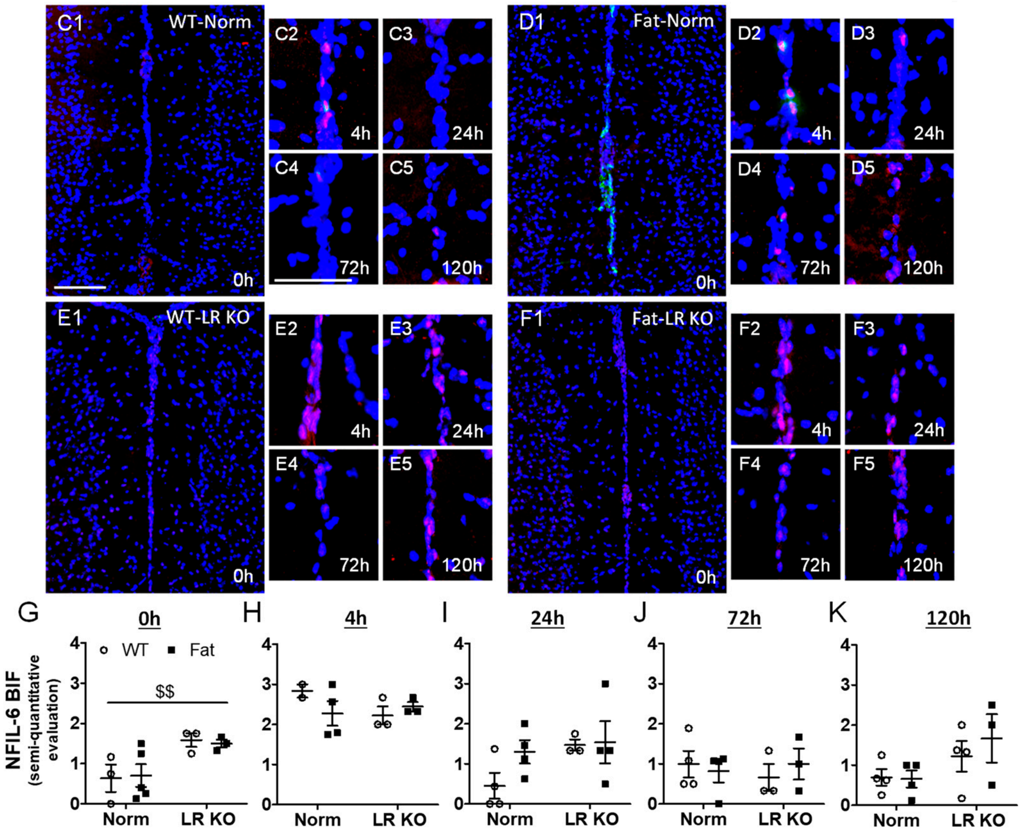

Figure S8: Intratracheal LPS-induced ARDS NF-IL6 immunoreactivity in the BIF in Norm compared to LR KO mice is significantly altered at 0 h p.i. Sections of the brain were analyzed at the level of the bifurcation (BIF; A) by semi-quantitative evaluation for nuclear factor interleukin 6 (NF-IL6; red, C-F) immunoreactivity on a scale ranging from 0-4 (B). Unmodified mice (Norm; C) were compared to mice deficient in the leukotriene B4 receptor (LR KO; E, F) bred on a wild-type (WT; C, E) or transgenic omega-3 (n-3) synthesizing *fat-1* (Fat; D, F) background received an intratracheal (i.t.) instillation with lipopolysaccharide (LPS, 10µg) and were sacrificed at 0 h, 4 h, 24 h, 72 h and 120 h p.i. WT- / Fat-Norm groups were compared to WT- / Fat-LR KO groups at each time point (G-K). At 0 h p.i. with LPS LR KO had altered NF-IL6 immunoreactivity compared to Norm. Von Willebrand factor (green; C, D) depicts brain vasculature. Myeloperoxidase (green; E, F) visualizes neutrophils. DAPI (blue; C-F) visualizes the surrounding tissue. Please note that data on WT-Norm and Fat-Norm controls is displayed again (Figure 5) for comparison. n = 2-5 per group. Statistics were only performed when n = >3 per group. $ main effect Norm vs. LR KO. Scale bar in C1 = 100 µm and is representative for C1, D1, E1 and F1; Scale bar in C4 = 50 µm and is representative for C2-5, D2-5, E2-5 and F2-5. **p<0.01.

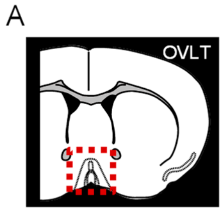

B

Neutrophils OVLT Means per 10× field	0 h	4 h	24 h	72 h	120 h
WT-Norm	0.4	6.3	1.0	1.0	0.4
Fat-Norm	0.2	2.0	1.6	1.5	0.2
WT-CR KO	5.0	4.1	2.4	2.3	2.9
Fat-CR KO	2.2	7.9	1.7	2.8	3.5

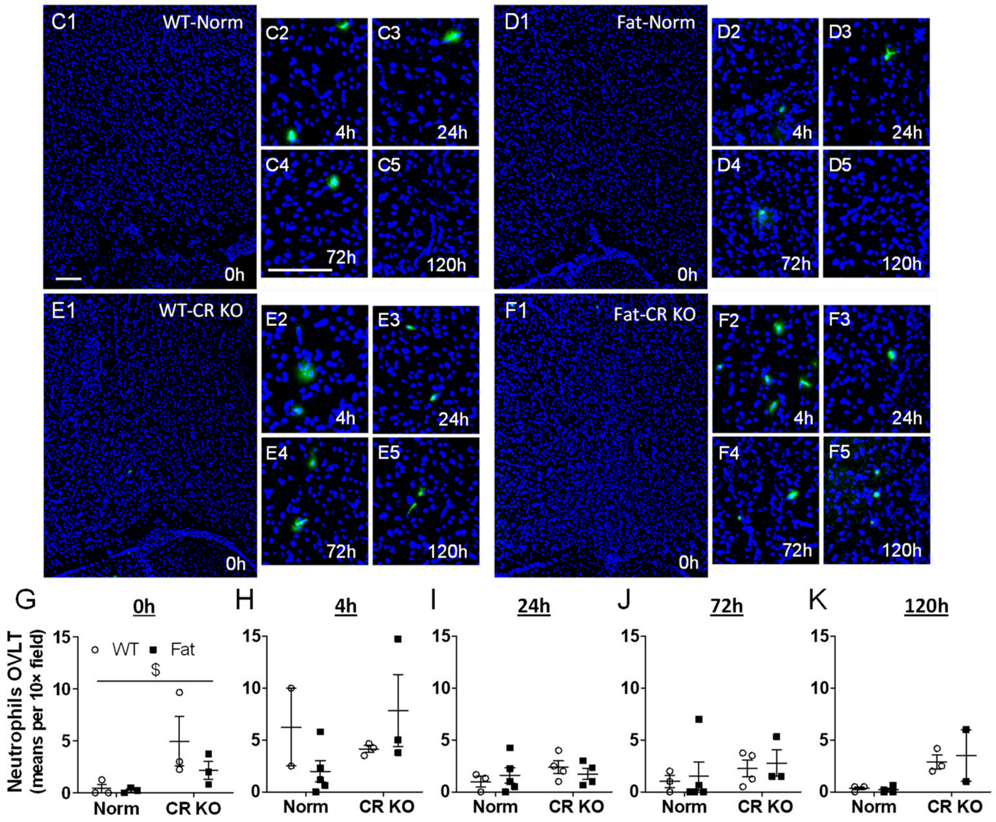

Figure S9. Intratracheal LPS-induced ARDS neutrophil recruitment at the level of the OVLT in Norm compared to CR KO mice is significantly altered at 0 h p.i. Sections of the brain were analyzed at the level of the vascular organ of lamina terminalis (OVLT; A) and neutrophils (myeloperoxidase, green; C-F) were counted per 10x field of view (B). Unmodified mice (Norm; C) were compared to mice deficient in the chemerin receptor 23 (CR KO; E) bred on a wild-type (WT; C, E) or transgenic omega-3 (n-3) synthesizing *fat-1* (Fat; D, F) background received an intratracheal (i.t.) instillation with lipopolysaccharide (LPS, 10μg) and were sacrificed at 0 h, 4 h, 24 h, 72 h and 120 h p.i. WT- / Fat-Norm groups were compared to WT- / Fat-CR KO groups at each time point (G-K). At 0 h p.i with LPS CR KO increased neutrophil recruitment compared to Norm regardless of n-3 enrichment. Intercellular adhesion molecule 1 (ICAM1; red, C-F). DAPI (blue; C-F) visualizes the surrounding tissue. n = 2-5 per group. Statistics were only performed when n = >3 per group. $ main effect Norm vs. CR KO. Scale bar in C1 = 100 μm and is representative for C1, D1, E1 and F1; Scale bar in C4 = 50 μm and is representative for C2-5, D2-5, E2-5 and F2-5. *p<0.05.

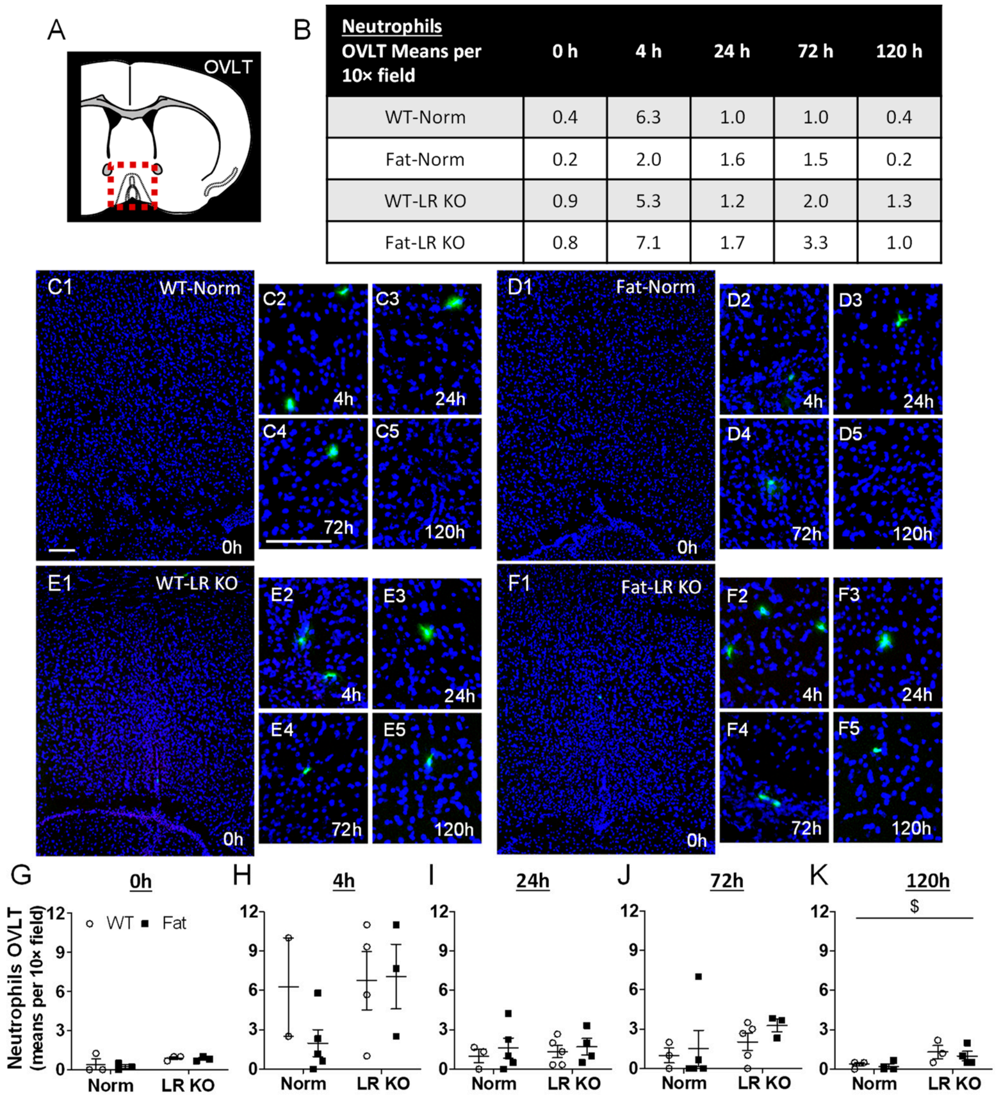

Neutrophils OVLT Means per 10× field	0 h	4 h	24 h	72 h	120 h
WT-Norm	0.4	6.3	1.0	1.0	0.4
Fat-Norm	0.2	2.0	1.6	1.5	0.2
WT-LR KO	0.9	5.3	1.2	2.0	1.3
Fat-LR KO	0.8	7.1	1.7	3.3	1.0

Figure S10: Intratracheal LPS-induced ARDS neutrophil recruitment to the OVLT in Norm compared to LR KO mice is significantly altered at 120 h p.i. Sections of the brain were analyzed at the level of the organum vasculosum lamina terminalis (OVLT; A) and neutrophils (green, C-F) were counted per 10x field of view (B). Unmodified mice (Norm; C) were compared to mice deficient in the leukotriene B4 receptor (LR KO; E, F) bred on a wild-type (WT; C, E) or transgenic omega (n-3) synthesizing *fat-1* (Fat; D, F) background received an intratracheal (i.t.) instillation with lipopolysaccharide (LPS, 10µg) and were sacrificed at 0 h, 4 h, 24 h, 72 h and 120 h p.i. WT- / Fat-Norm groups were compared to WT- / Fat-LR KO groups at each time point (G-K). At 120 h p.i. with LPS LR KO increased neutrophil recruitment compared to Norm regardless of n-3 enrichment. Intercellular adhesion molecule 1 (ICAM1; red, C, D). DAPI (blue; C-F) visualizes the surrounding tissue. Please note that data on WT-Norm and Fat-Norm controls is displayed again (Figure S9) for comparison. n = 2-5 per group. Statistics were only performed when n = >3 per group. $ main effect Norm vs. LR KO. Scale bar in C1 = 100 µm and is representative for C1, D1, E1 and F1; Scale bar in C4 = 50 µm and is representative for C2-5, D2-5, E2-5 and F2-5. *p<0.05.

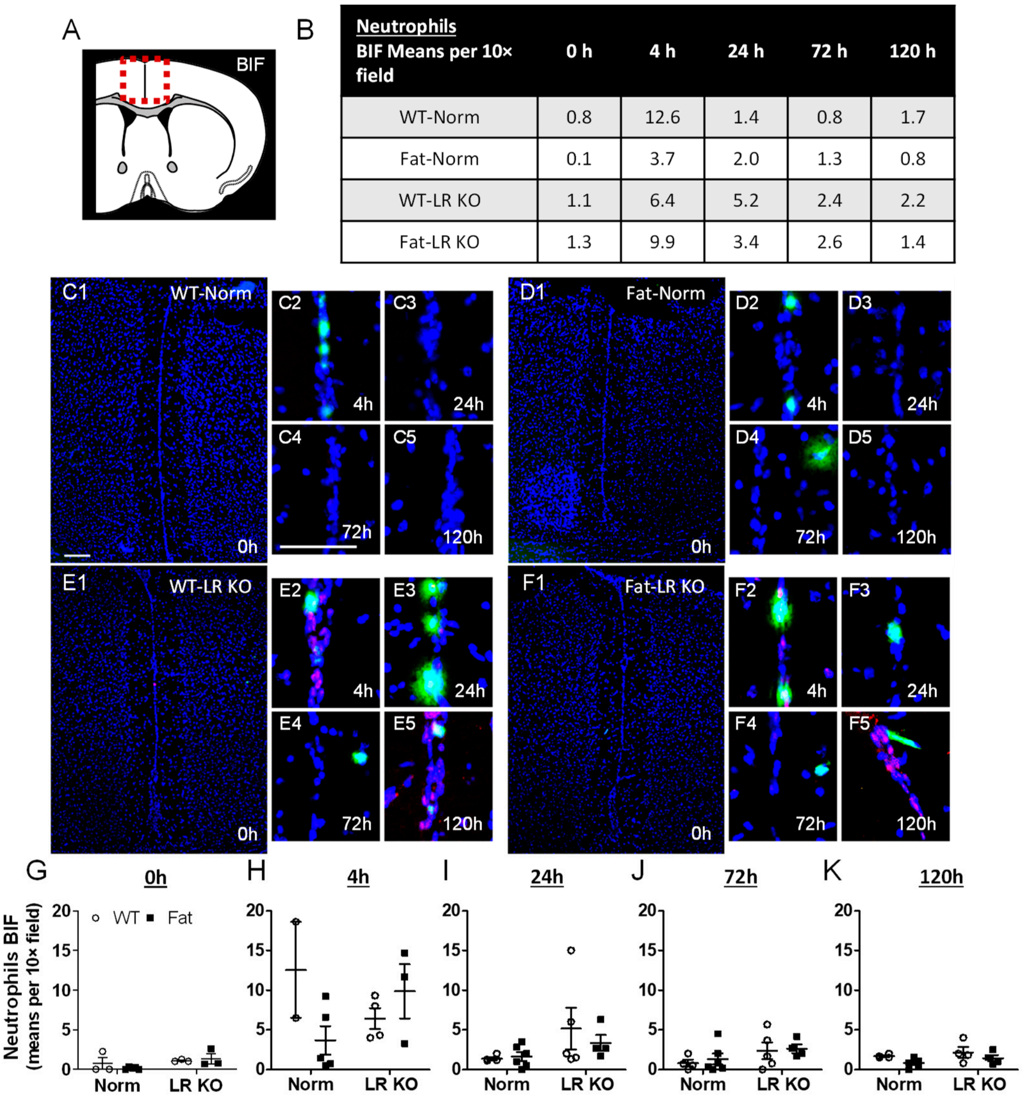

Neutrophils BIF Means per 10× field	0 h	4 h	24 h	72 h	120 h
WT-Norm	0.8	12.6	1.4	0.8	1.7
Fat-Norm	0.1	3.7	2.0	1.3	0.8
WT-LR KO	1.1	6.4	5.2	2.4	2.2
Fat-LR KO	1.3	9.9	3.4	2.6	1.4

Figure S11: Intratracheal LPS-induced ARDS neutrophil recruitment to the BIF in Norm compared to LR KO mice is not significantly altered at a specific time point. Sections of the brain were analyzed at the level of the bifurcation (BIF; A) and neutrophils (green, C-F) were counted per 10x field of view (B). Unmodified mice (Norm; C) were compared to mice deficient in the leukotriene B4 receptor (LR KO; E, F) bred on a wild-type (WT; C, E) or transgenic omega (n-3) synthesizing *fat-1* (Fat; D, F) background received an intratracheal (i.t.) instillation with lipopolysaccharide (LPS, 10µg) and were sacrificed at 0 h, 4 h, 24 h, 72 h and 120 h p.i. WT- / Fat-Norm groups were compared to WT- / Fat-LR KO groups at each time point (G-K). No differences were observed between groups. Intercellular adhesion molecule 1 (ICAM1; red, C, D). Nuclear factor interleukin 6 (NF-IL6; red, E, F). DAPI (blue; C-F) visualizes the surrounding tissue. Please note that data on WT-Norm and Fat-Norm controls is displayed again (Figure 8) for comparison. n = 2-6 per group. Statistics were only performed when n = >3 per group. Scale bar in C1 = 100 µm and is representative for C1, D1, E1 and F1; Scale bar in C4 = 50 µm and is representative for C2-5, D2-5, E2-5 and F2-5.

Table S1: The p-values for main effects (M.E.) of RvE1 receptors and omega-3 polyunsaturated fatty-acids (n-3 PUFAs) on inflammatory mediators between unmodified (Norm) mice and chemerin receptor 23 (CR) or leukotriene B4 receptor (LR) knock out (KO) mice in the lung at 0 h, 24 h and 72 h p.i.

Time	M.E.	Group: Norm vs.	IL-17	IL-1β	TNFα	GM-CSF	CXCL1	CXCL5	IL-10
**0 h**	**RvE1 receptor**	CR KO							0.0080
		LR KO	0.0064						
	**n-3 PUFAs**	CR KO				0.0120			
		LR KO							
**24 h**	**RvE1 receptor**	CR KO	0.0334						0.0017
		LR KO							0.0372
	**n-3 PUFAs**	CR KO					0.0321		
		LR KO						0.0374	0.0200
**72 h**	**RvE1 receptor**	CR KO	0.0465	0.0176	0.0477	0.0148		0.0098	0.0008
		LR KO							
	**n-3 PUFAs**	CR KO							
		LR KO							

Table S2: The p-values for main effects (M.E.) of omega-3 polyunsaturated fatty-acids (n-3 PUFAs) and RvE1 receptors on inflammatory mediators between unmodified (Norm) mice and chemerin receptor 23 (CR) or leukotriene B4 receptor (LR) knock out (KO) mice in the liver at 24 h p.i.

Time	M.E.	Group: Norm vs.	CCL5	IL-17	IL-10
**24 h**	**RvE1 receptor**	CR KO			
		LR KO			0.0065
	**n-3 PUFAs**	CR KO	0.0355	0.0281	0.0347
		LR KO		0.0388	

Table S3: The p-values for main effects (M.E.) of omega-3 polyunsaturated fatty-acids (n-3 PUFAs) and RvE1 receptors on inflammatory mediators between unmodified (Norm) mice and chemerin receptor 23 (CR) or leukotriene B4 receptor (LR) knock out (KO) mice in the hypothalamus at 0 h, 24 h and 72 h p.i.

Time	M.E.	Group: Norm vs.	Cat	SOD1	SOD2	NOS2	TFAM	Elane	CXCL1	IL-1β	IL-1ra	NF-IL6	PGC1α	GFAP
0 h	RvE1 receptor	CR KO				0.0172			0.0261					0.011
		LR KO												
	n-3 PUFAs	CR KO									0.0347	0.0380		
		LR KO			0.0005		0.0268			0.0286			0.0280	
24 h	RvE1 receptor	CR KO				0.0275			0.0152	0.0268	0.0385			
		LR KO						0.0440	0.0380	0.0346				
	n-3 PUFAs	CR KO					0.0487		0.0275	0.0126	0.0241			
		LR KO							0.0118	0.0044	0.0156			
72 h	RvE1 receptor	CR KO				0.0008								
		LR KO												
	n-3 PUFAs	CR KO	0.0077	0.0178	0.0463	0.0029	0.0404							
		LR KO												

Table S4: The main effects (M.E.) of RvE1 receptors and omega-3 polyunsaturated fatty-acids (n-3 PUFAs) on lipid mediators between unmodified (Norm) mice and chemerin receptor 23 (CR) or leukotriene B4 receptor (LR) knock out (KO) mice in the lung at 0 h, 24 h and 72 h p.i.

Time	M.E.	Group: Norm vs.	LTB_4	EPA	18-HEPE	RvE1
**0 h**	**RvE1 receptor**	CR KO	<0.0001	<0.0001		
		LR KO		<0.0001		
	**n-3 PUFAs**	CR KO		<0.0001	<0.0001	
		LR KO	0.0006	<0.0001		
**24 h**	**RvE1 receptor**	CR KO		0.0132		
		LR KO	<0.0001	0.0001		
	**n-3 PUFAs**	CR KO			<0.0001	<0.0001
		LR KO			<0.0001	<0.0001
**72 h**	**RvE1 receptor**	CR KO	<0.0001			
		LR KO	0.0023	0.0024		
	**n-3 PUFAs**	CR KO			<0.0001	<0.0001
		LR KO	0.0018		<0.0001	<0.0001

Table S5: The main effects (M.E.) of RvE1 receptors and omega-3 polyunsaturated fatty-acids (n-3 PUFAs) on lipid mediators between unmodified (Norm) mice and chemerin receptor 23 (CR) or leukotriene B4 receptor (LR) knock out (KO) mice in the brain at 0 h, 24 h and 72 h p.i.

Time	M.E.	Group: Norm vs.	AA	LTB_4	EPA	18-HEPE	RvE1	DHA	17(S)-HDHA	NPD1+ PDX	RvD1	RvD2	14(S)-HDHAα	Mar1
	**RvE1 receptor**	CR KO		<0.0001		0.0005			<0.0001	0.0003			<0.0001	<0.0001
		LR KO	0.0029	<0.0001	0.0006			0.0027	<0.0001	<0.0001			<0.0001	<0.0001
	**n-3 FAs**	CR KO	0.0004		<0.0001			0.0001		<0.0001				<0.0001
**0 h**		LR KO	0.0004	0.0270	0.0002	0.0084		0.0002		<0.0001			0.0131	<0.0001
	**RvE1 receptor**	CR KO												
		LR KO	0.0011		0.0013			0.0043	<0.0001	0.0033			<0.0001	**0.0002**
**24 h**	**n-3 FAs**	CR KO			0.0003	0.0032				0.0498				
		LR KO						0.0071		<0.0001			<0.0001	**<0.0001**
	**RvE1 receptor**	CR KO				0.0004								
		LR KO							<0.0001				0.0003	
**72 h**	**n-3 FAs**	CR KO	0.0253		0.0090			0.0030		<0.0001			0.0105	
		LR KO				0.0002								**<0.0001**

Table S6: Antibodies for inflammatory markers in the brain

Antigen	Specification	Dilution	Product information	Manufacturer
**primary antibodies**				
ICAM-1	Polyclonal IgG, goat	1:500	sc-1511	Santa Cruz Biotechnology, Santa Cruz, CA, USA
MPO	Polyclonal IgG, rabbit	1:600	A0398	Dako Denmark A/S, Glostrup, Denmark
MPO	Polyclonal IgG, goat	1:200	AF3667	R & D Systems Biotech Co., Minneapolis, MN, USA
NF-IL6	Polyclonal IgG, rabbit	1:5000 / 1:1000	sc-150	Santa Cruz Biotechnology, Santa Cruz, CA, USA
vWF	Polyclonal IgG, sheep	1:3000	SARTW-IG	Affinity Biologicals, Ancaster, ON, Canada
GFAP	Polyclonal IgG, guinea pig	1:500	173 004	Synaptic Systems GmbH, Göttingen, Germany
**secondary antibodies**				
Alexa 488, donkey	Anti-sheep	1:500	A11015	Life Technologies, Carlsbad, CA, USA
Cy3, donkey	Anti-rabbit	1:600	711-165-152	Jackson Immuno Research Europe Ltd., Newmarket, UK
Alexa 647, donkey	Anti-guinea pig	1:200	706-605-148	Dianova® GmbH, Hamburg, Germany

Table S7: Mass spectrometer settings for the Bruker Daltonik amaZon SL.

	**Settings**
General	MS stage: MS/MS (MS^2), MRM "on"<br>Polarity: negative<br>Trap: ICC "on", Target "35.000", Max. Accu Time "50ms",<br>Scan "70 to 700m/z", Averages "3"<br>Rolling Averaging: No. "3"
Mode	Scan Mode: Ultra Scan
Source	Capillary: 3600V, End Plate Offset: 500V,<br>Nebulizer: 4.0psi, Dry Gas: 2.0l/min, Dry Temp: 80°C
MRM	MS/MS: Isolation "on", width "1.5", Reaction "on", Cut-Off Selection "default",<br>Smart Frag "Enhanced" (for all precursors)<br>Segment Limit 1 (0 - 9.5min): Precursor 349, 347, 375, 359<br>Segment Limit 2 (9.5 - 45min): Precursor 335, 339, 317, 343, 359, 375<br>Segment Limit 3 (45 - 110min): Precursor 301, 306, 327, 303, 314

TableS8: Compounds, Retention Times and EIC MS^2 trace definitions.

Compound	Retention Time [min]	EIC MS^2
RvE1	6,7	291; 269; 205; 195; 161 - MS2(349)
RvD2	7,6	277; 259; 241; 233; 215; 141 - MS2(375)
RvD1	8,1	277; 259; 241; 233; 215; 141 - MS2(375)
NPD1 / PDX (coelution)	10,3	261; 245; 217; 206; 153 - MS2(359)
Mar1	10,6	250; 221; 177 - MS2(359)
LTB_4-d_4 (Internal Standard - 1)	11,2	197 - MS2(339)
LTB_4	11,2	255; 195; 181; 129 - MS2(335)
18-HEPE	15,9	259; 215 - MS2(317)
17(S)-HDHA	21,9	245; 201 - MS2(343)
14(S)-HDHA	23,3	205; 161 - MS2(343)
EPA-d_5 (Internal Standard - 2)	61	262; 208 - MS2(306)
EPA	62,1	257; 203 - MS2(301)
DHA	88,6	283; 229 - MS2(327)
AA-d_{11} (Internal Standard - 3)	96,3	270; 216 - MS2(314)
AA	99,2	259; 205 - MS2(303)
